# Cardio-oncology: conflicting priorities of anticancer treatment and cardiovascular outcome

**DOI:** 10.1007/s00392-018-1202-x

**Published:** 2018-02-16

**Authors:** Lisa M. Tilemann, Markus B. Heckmann, Hugo A. Katus, Lorenz H. Lehmann, Oliver J. Müller

**Affiliations:** 1 Abteilung für Kardiologie, Pneumologie und Angiologie, Universitätsklinikum Heidelberg, Im Neuenheimer Feld 410, 69120 Heidelberg, Germany; 2 Deutsches Zentrum für Herz-Kreislauf-Forschung, Standort Heidelberg, Mannheim, Germany; 3 Present Address: Department of Internal Medicine III, University of Kiel, Arnold-Heller-Str. 3, 24105 Kiel, Germany

**Keywords:** Cardiotoxicity, Side effects, Cardio-oncology, Onco-cardiology

## Abstract

**Background:**

This article about the emerging field of cardio-oncology highlights typical side effects of oncological therapies in the cardiovascular system, cardiovascular complications of malignancies itself, and potential preventive or therapeutic modalities.

**Methods:**

We performed a selective literature search in PubMed until September 2016.

**Results:**

Cardiovascular events in cancer patients can be frequently attributed to oncological therapies or to the underlying malignancy itself. Furthermore, many patients with cancer have pre-existing cardiovascular diseases that can be aggravated by the malignancy or its therapy. Cardiovascular abnormalities in oncological patients comprise a broad spectrum from alterations in electrophysiological, laboratory or imaging tests to the occurrence of thromboembolic, ischemic or rhythmological events and the impairment of left ventricular function or manifest heart failure.

**Discussion:**

A close interdisciplinary collaboration between oncologists and cardiologists/angiologists as well as an increased awareness of potential cardiovascular complications could improve clinical care of cancer patients and provides a basis for an improved understanding of underlying mechanisms of cardiovascular morbidity.

## Introduction

**Fig. 1:**
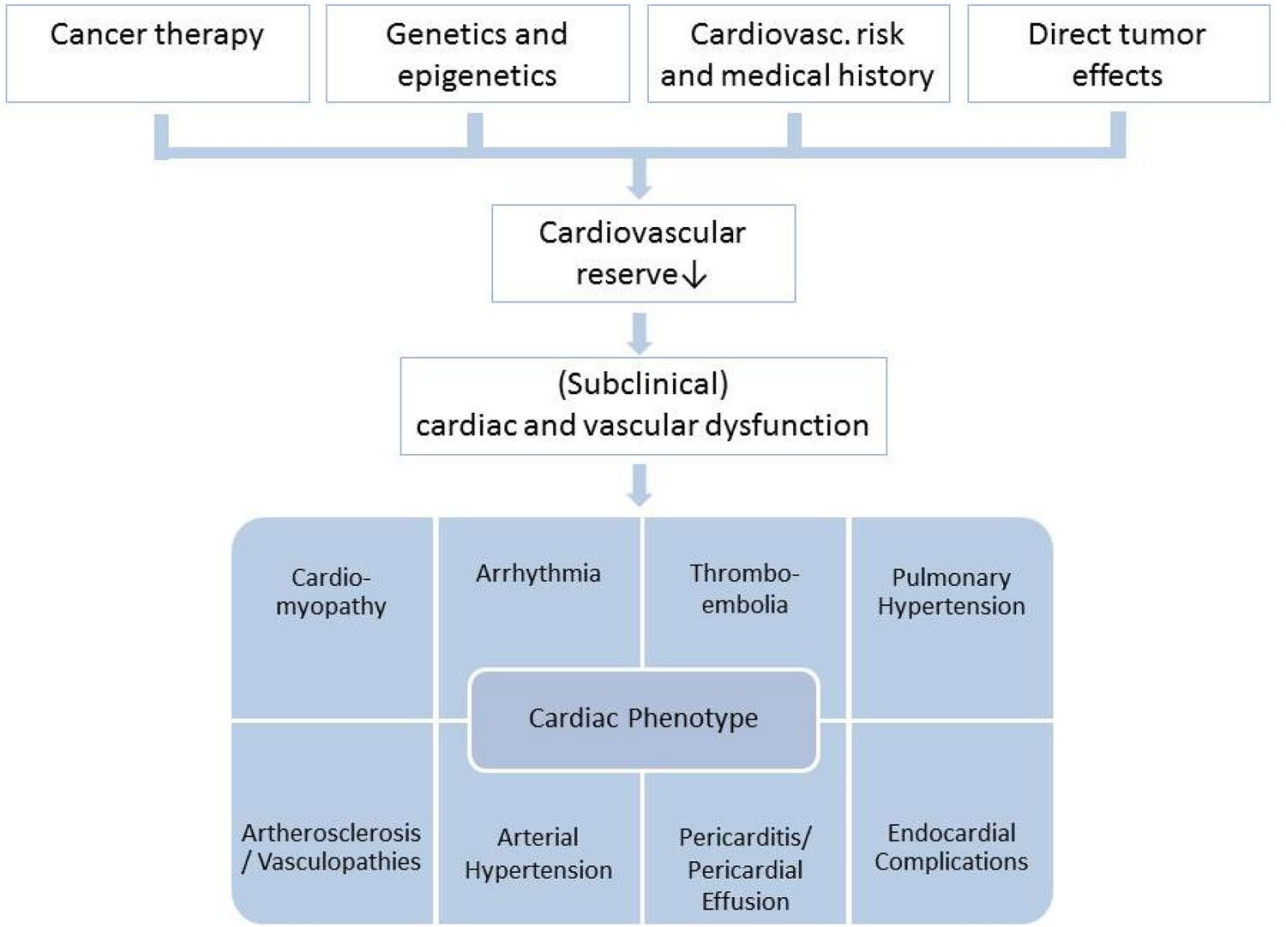
Multifactorial cardiovascular risk model for the occurrence of cardiotoxic effects in oncologic patients

Scientific advances have led to a steady increase in survival rates of many oncologic entities within in the last decades. With more patients surviving their cancer, long-term side effects of oncologic therapies warrant consideration. In particular, cardiotoxic side effects following cancer therapy account for a relevant decrease in quality of life and mortality. On the other hand, a hasty withdrawal or de-escalation of oncologic therapy with the first appearance of cardiovascular side effects might worsen long-term prognosis. Combined chemotherapy or adjuvant radiotherapy can have synergistic or additive effects on the risk for cardiac complications and increase the cardiotoxic potential [1]. Cardiotoxicity of a certain therapy is difficult to predict for the individual patient as multiple factors contribute to an unknown extent (Fig. 1).

Patients with increased cardiovascular risk profiles are more prone to chemotherapy-associated cardiac dysfunction and deterioration of pre-existing cardiovascular diseases [2, 3]. However, even when adjusting for cardiovascular risk, there is still a high variability regarding the individual susceptibility to cardiotoxic effects. Subsequently, genetics and genotyping have gained more attention within the last decade [4, 5]. Aside from iatrogenic cardiotoxicity, there is also some evidence for the cancer itself affecting cardiac function and exercise performance, measured by peak oxygen consumption [6]. Accordingly, different preclinical models have described cancer-related myocardial atrophy, cardiac remodeling and cellular dysfunction, which are summarized as cardiac cachexia [7].

Distinguishing tumor associated or iatrogenic cardiotoxicity from inherent cardiac diseases is one of the tasks of the still young field of cardio-oncology. Further tasks include the interdisciplinary management of patients with cardiac tumors or metastases, myocardial, endocardial or pericardial complications, therapy-induced ischemia or arrhythmias, vascular complications, and venous thromboembolism. This review gives an overview of major cardiovascular implications of oncologic diseases and therapies.

## Myocardial dysfunction

Myocardial dysfunction is a frequent complication of various oncologic therapies. Although there is no generally accepted definition of therapy-induced cardiotoxicity, echocardiographic assessment of left ventricular ejection fraction (LVEF) is still the most commonly used parameter to diagnose toxic cardiomyopathy [8, 9]. A symptomatic or asymptomatic decrease in LVEF of more than 10% to less than 50% is considered clinically relevant [10, 11]. In patients with symptoms or signs of heart failure but with preserved ejection fraction, echocardiographic assessment of diastolic function should be performed and natriuretic peptide should be measured [11]. Recent data do also suggest that strain and strain rate analysis might be important alone or in combination with biomarkers, to assess the individual course of cardiotoxicity in oncological patients [12].

In recent years, two distinct forms of cardiotoxicity have been described—a dose-dependent, cumulative, mostly irreversible form and a reversible, dose-independent type; see Table 1. The dose-independent type of cardiotoxicity is usually not associated with structural damage of cardiomyocytes, thus, chemotherapy can often be resumed after a short pause or in mild forms be continued [13, 14].

**Table 1:** Characteristics of type-1 and type-2-cardiotoxicity. Modified from [15]

Drugs	Histopathology	Reversibility	Dose-dependency	Prognostic value
Doxorubicin Daunorubicin Epirubicin Idarubicin Mitoxantrone Cyclophosphamide	Apoptosis, necrosis-damaged sarcomeres	No (partially responsive to heart failure therapy)	Yes	Associated with increased mortality
Trastuzumab Sunitinib Lapatinib Imatinib	Myocyte dysfunction	Yes (in most cases)	No	Not associated with increased mortality

This classification might facilitate therapeutic decisions, but has limitations when it comes to the individual patient’s prognosis regarding cardiac function. In addition, chemotherapeutic drugs are often applied as combination therapies or are administered sequentially. Thus, the interaction of diverse toxic effects makes it difficult to distinguish between different pathomechanisms.

The best-known example of cardiotoxicity is anthracycline-induced cardiomyopathy. Similar effects, however, have also been shown for other chemotherapeutic agents like cyclophosphamide [16]. Anthracyclines may infrequently cause acute, dose-independent, and reversible cardiac dysfunction directly following infusion, which is to be distinguished from dose-dependent chronic cardiomyopathy. Reactive oxygen species and oxidative stress seem to play an important role in its pathomechanism leading to a decrease in myocardial mass, cardiac remodeling and ultimately cardiac dysfunction [17, 18]. Approximately 5% of patients treated with a cumulative dose of 400–450 mg/m^2^ doxorubicin develop heart failure; the proportion increases to 10% in elderly patients [19, 20]. Once a cumulative dose of more than 300 mg/m^2^ doxorubicin has been administered, the addition of dexrazoxane can be considered to decrease toxicity [17, 21]. However, a general recommendation for dexrazoxane cannot be given as it might decrease the efficacy of anthracycline therapy. Other cardioprotective measures include the usage of liposomal formulas and prolonged infusions (more than 30 min). However, a potential decrease in efficacy is also assumed for these measures.

Heart failure therapy in oncologic patients follows the same principles as in other patients and has shown some reversibility even in chronic anthracycline-induced cardiomyopathy when initiated early [22]. In contrast, preventive heart failure therapy accompanying chemotherapy or radiation in the absence of heart failure is discussed controversially [23–25]. There might be, however, beneficial effects in high-risk populations, namely, patients undergoing high-dose chemotherapy with anthracyclines [26, 27]. Accordingly, current recommendations consider a preventive cardioprotective therapy for patients at high-risk cardiac complications [10].

**Fig. 2:**
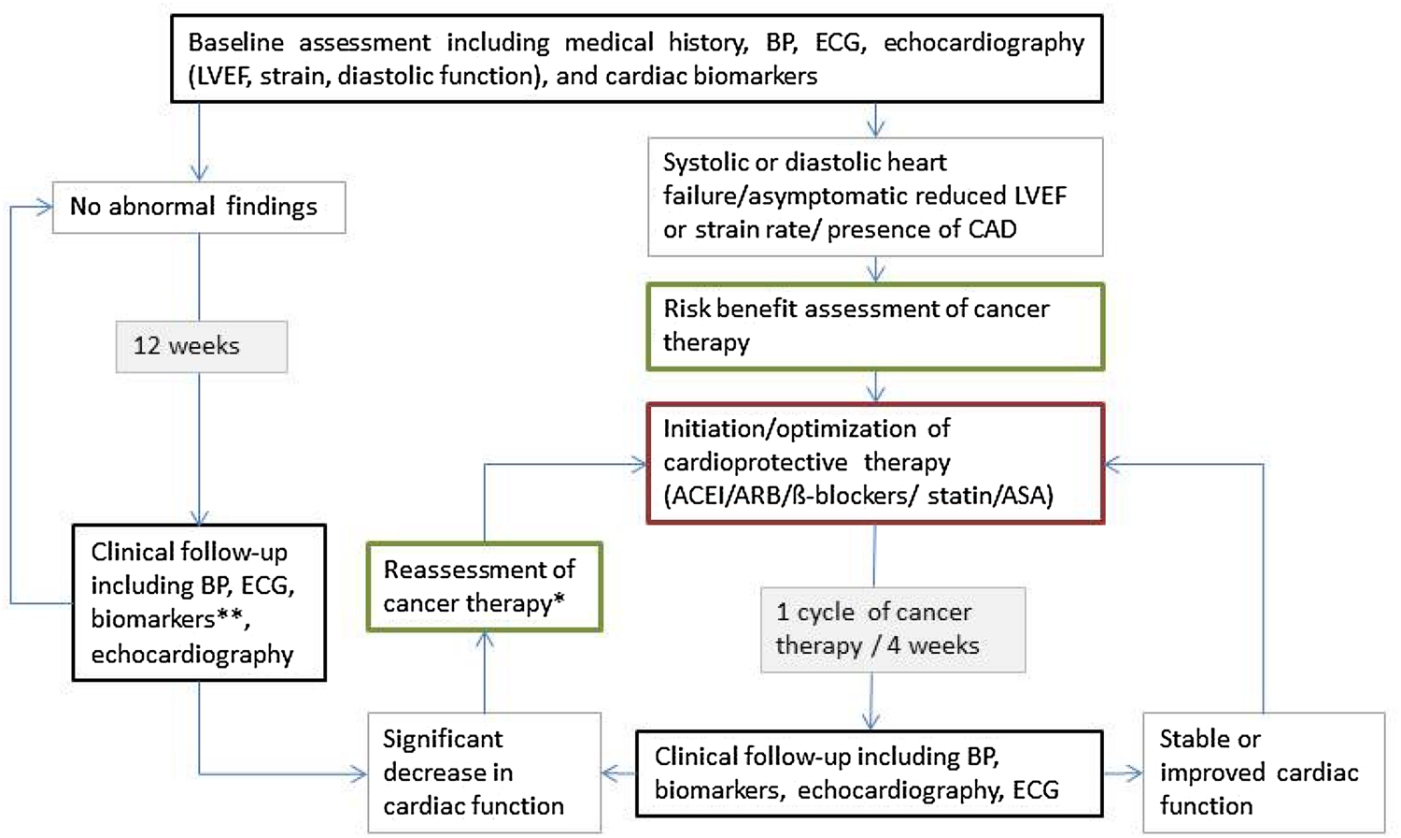
Cardiac monitoring of patients during potentially cardiotoxic cancer therapy. *Reassessment with discontinuation of cardiotoxic therapy vs. dose reduction vs. change of chemotherapeutic drug vs. unmodified continuation of oncologic treatment. **Optional. *BP* blood pressure measurement, *ECG* electrocardiogram, *LVEF* left ventricular ejection fraction, *ACEI* angiotensin converting enzyme inhibitors, *ARB* angiotensin receptor blockers, *ASA* acetylsalicylic acid

In order to monitor patients with pre-existing cardiac dysfunction as well as to identify patients with an increased cardiac risk, echocardiography, ECG, and measurement of biomarkers such as natriuretic peptide (BNP or nt-proBNP) or (high-sensitive) troponin are recommended (Fig. 2). The value of routine measurements of cardiac biomarkers to monitor asymptomatic patients without any pre-existing cardiac conditions is still uncertain as the clinical significance of minor changes is yet to be elucidated. With this in mind, cardiac biomarkers can still be useful to track minor subclinical changes in cardiac function, which might influence decisions on oncologic treatment strategies or preventive measures [10].

## Venous thromboembolism

Venous thromboembolism (VTE) is a frequent complication in cancer patients. The risk for VTE is 4–7 times increased in cancer patients compared to persons without malignancy [28]. The risk for VTE depends on the type of malignancy, the stage of disease, the oncologic treatment, and patient-specific factors (Table 2). Suspected VTE in cancer patients is usually clarified by diagnostic imaging, i.e., compression ultrasound for deep vein thrombosis, and CT-angiography for pulmonary embolism, respectively [29]. d-dimers are often unspecifically increased and should not be used to rule out VTE in cancer patients [29]. Low molecular heparins (LMH) are first-line therapy during the first 3–6 months after diagnosis and are usually followed by long-term anticoagulation as long as the cancer is active [29]. This also applies to catheter-associated intravenous thrombosis as long as the catheter is functional, in use, and shows no signs of bacterial infection.

**Table 2:** Thromboembolic risk factors in oncologic patients

Risk	Description
Individual risk	Age [30] Positive anamnesis or family history for VTE [31] Hereditary thrombophilia (e.g., factor-V-Leiden mutation) [32] Life style (obesity, lack of physical exercise, smoking) [30, 33] Oral contraceptive therapy Ethnicity (increased risk in Afro-Americans) [34]
Risk of tumor entity and stage	Tumor entity (high risk with pancreatic, gastric, renal, lung or ovarian cancer as well as lymphoma and malign brain tumors) [30, 32–34] Metastasized disease stage [30, 32, 34] First 3–6 months following diagnosis [32, 34] Thrombocytosis before initiation of chemotherapy [33] Increased inflammatory markers Increased d-dimers
Oncologic therapy	Surgery [35] Radiation Chemotherapy [28, 31] Hormone therapy [36] Immune therapy Antiangiogenic therapy [37, 38]
Other risk factors	Blood transfusions Drugs stimulating erythropoiesis (e.g., erythropoietin) [33] Central venous catheter (e.g., port systems) [39] Immobilization, hospitalization [30, 31] Dehydration due to vomiting or diarrhea

An increased incidence of VTE has been described for cisplatin, bevacizumab (angiogenesis inhibitor), tamoxifen (selective estrogen receptor blocker), and sunitinib and sorafenib (tyrosine kinase inhibitors) [36–38, 40]. Routine prophylactic anticoagulation, however, is not recommended in cancer patients, even when the above-mentioned drugs are administered [29, 41]. Aside from the lack of clinical data, the increased risk for major bleedings and the comparatively low incidence of VTEs in an outpatient setting support this recommendation [42]. In contrast, the benefit of a prolonged thrombosis prophylaxis following major abdominal or pelvic tumor surgery is well-described [35]. Furthermore, patients suffering from multiple myeloma and who are treated with lenalidomide or thalidomide should routinely receive anticoagulation therapy [41, 43, 44]. Prophylactic therapy is also recommended for hospitalized cancer patients with active disease [41]. Other situations (e.g., bed-ridden patients) need individual decisions.

## Endocardial complications/endocarditis

### Thrombotic endocarditis

Thrombotic endocarditis (TE) is a form of non-bacterial endocarditis affecting mainly patients with advanced cancer stages [45]. Often patients remain asymptomatic as long as no arterial embolization occurs. Distinguishing TE form infectious endocarditis (IE) can be challenging. A disseminated embolic distribution pattern and low inflammatory markers are indicative of TE [46]. TE is also likely if echocardiographic criteria of endocarditis are met but blood cultures remain negative or if there is no clinical response to antibiotic therapy. Other causes of blood culture-negative endocarditis should be ruled out. Vegetations are most commonly located to left ventricular valves, specifically at the ventricular side of the aortic valve and atrial side of the mitral valve [47]. Surgical therapy is rarely needed. Besides treating the underlying disease, systemic anticoagulation therapy is indicated to prevent thromboembolic complications [48, 49].

### Infectious endocarditis

Intravascular foreign bodies (e.g., catheter-systems) as well as an altered immune response enable the development of IE in cancer patients. Due to a lack of data regarding the incidence of IE in cancer patients, IE prophylaxis, diagnosis, and therapy follows the same guidelines as in other patients with IE [50]. Every diagnosed catheter-associated infection in combination with at least one positive peripheral blood culture should be considered for further evaluation by transthoracic echocardiography (TTE), and transesophageal echocardiography (TEE) if indicated. Lack of adequate clinical response (e.g., persistent fever or persistent positive blood cultures) or the presence of additional risk factors (e.g., artificial heart valves, pace-maker or implantable cardioverter defibrillator) warrant echocardiographic evaluation.

As cancer patients are prone to IE, IE might also be the first complication of undiagnosed cancer. IE is associated with an increased risk for cancer, in particular hematologic or intraabdominal malignancies [51]. For example, due to a strong association with colorectal cancer, guidelines recommend to perform a colonoscopy if *S. bovis* or *S. gallolyticus* has been detected in blood cultures [50].

### Hedinger’s syndrome

Paraneoplastic processes might also favor valvulopathy. Patients suffering from certain types of neuroendocrine tumors, carcinoids, gradually develop right ventricular endocardial fibrosis. This paraneoplastic process eventually leads to Hedinger’s syndrome which is characterized by a degeneration and restriction of the tricuspid and pulmonary valve. Therapy focuses primarily on the treatment of the underlying disease [52]. The valvulopathy and subsequent right ventricular failure is treated primarily with diuretics and in some cases with surgical valve replacement [53].

## Pericardial complications

A newly diagnosed pericardial effusion might represent the first sign of an underlying malignancy. Cytological analysis of the pericardial effusion and peri-/epicardial biopsies should be pursued [54–56]. Almost 2/3 of pericardial effusions in cancer patients, however, are not caused by direct tumor infiltration, but are due to paraneoplastic processes, former radiation, or due to an opportunistic infection [56]. If cardiac tamponade is imminent, pericardiocentesis should be performed promptly. Pericardial effusions often re-occur in these patients and are difficult to manage. Radiotherapy might likely lead to a reduction of the associated pericardial effusion in the presence of radiation sensitive tumors. However, radiotherapy itself is also associated with pericardial effusion although the use of modern protocols has reduced its occurrence [57]. Pericardial fenestration might provide symptomatic control with frequently recurring pericardial effusions [54, 58]. In some cases, intrapericardial application of cytostatic or sclerosing agents might represent the only feasible therapy [54, 56, 59, 60]. As pericardial involvement often implies a palliative stage, control of symptoms and improving quality of life should be the primary focus of any therapy.

## Arterial hypertension

Arterial hypertension has been associated with various chemotherapeutic agents [61]. Drugs modifying the vascular endothelial growth factor (VEGF) pathway frequently increase systemic blood pressure [62]. Tyrosine kinase inhibitors are also associated with an increase in systemic blood pressure, which occurs often as early as a few hours after initiation of treatment [62]. A disturbance in endothelial function and alterations on the capillary level are likely pathomechanisms linked to this effect [63]. Patients receiving chemotherapeutic agents associated with arterial hypertension should be screened on a weekly basis for arterial hypertension during the first cycle [61]. The interval can be prolonged to 2 or 3 weeks in time [61]. Discontinuation of the chemotherapeutic drug should be considered in patients with a hypertensive crisis or a persistent systolic blood pressure over 180 mmHg despite adequate antihypertensive therapy [61]. In general, blood pressure therapy in cancer patients follows the same principles as in other patients with the exception that diuretics should be used with particular caution because of potential electrolyte impairments in cancer patients. In addition, drug interactions require particular care in choosing the most appropriate antihypertensive therapy as some chemotherapeutic and antihypertensive drugs share the same metabolic pathways.

## Arteriosclerosis and complications

Atherosclerotic processes usually evolve over several years before symptoms occur. This latency might contribute to the fact that the effect of chemotherapeutic agents on blood vessels is still not well understood. Furthermore, cancer and atherosclerosis share the most potent risk factor: smoking [64]. The co-prevalence of different cancers and clinical manifestations of atherosclerosis complicates the distinction between toxic side effects of chemotherapy and pre-existing cardiovascular risk. However, for some chemotherapeutic agents, such as cisplatin, bleomycin, and etoposide, an increased long-term risk for cardiovascular and atherosclerotic complications has been confirmed [65, 66]. These long-term effects have to be distinguished from acute vascular events caused by arterial thrombosis, which might lead to thrombotic occlusion of coronary vessels in the absence of coronary artery disease [67]. Substances such as 5-FU and capecitabine, or paclitaxel, gemcitabine, rituximab, and sorafenib have been linked to vascular spasm and Raynaud phenomenon, angina pectoris, and even myocardial infarction [68–70].

While more clinical research is needed to understand long-term arterial complications of chemotherapy, long-term effects of radiotherapy on blood vessels is comparatively well studied. Inflammatory processes lead to atherosclerotic lesions in irradiated vessels [71]. Patients with pre-existing coronary artery disease or a high cardiovascular risk profile are particularly prone to cardiovascular events following radiation [71]. Accordingly, adults that underwent radiotherapy of the chest during childhood have a significantly elevated risk for myocardial infarction, even in the absence of other cardiovascular risk factors [72, 73]. Radiation of the head and neck increases the cerebrovascular event rate and radiation of the pelvis might trigger the development of peripheral artery disease [74]. Apart from a few exceptions, there are no differences with regard to therapy and secondary prophylaxis when compared to non-irradiated patients [75]. As an example, carotid artery stenting might be preferred over carotid endarterectomy in patients with prior radical neck dissection and radiation therapy who require treatment for carotid stenosis, because of an increased rate of wound complications and kidney failure in patients undergoing surgery [76].

## Pulmonary hypertension

The exact prevalence of pulmonary hypertension (PH) in cancer patients is unknown. Initial, mostly exercise-induced symptoms such as dyspnea, fatigue, dizziness, and infrequently angina are unspecific. In addition to chronic thrombembolia, PH is frequently caused by drug-induced chemotoxicity. Especially alkylating agents (e.g., cyclophosphamide) have been associated with PH. Furthermore, radiotherapy of the chest has been associated with veno-occlusive PH. Most often, however, the development of PH in cancer patients has a multifactorial etiology. Post-capillary PH due to left ventricular dysfunction should be distinguished from pulmonary tissue disease or chronic thromboembolic PH as treatment and prognosis vary. Diagnostic workup should take into account the patient’s individual prognosis [77]. Echocardiography often provides the first clues for the presence of PH. To diagnose PH and distinguish post-capillary from other forms of PH, right heart catheterization should be performed. In the presence of right heart failure and volume overload, diuretics provide symptomatic relieve. PH therapy depends on the underlying processes and should follow current guidelines [77]. However, adaptations might be necessary due to altered life expectancy and drug interactions.

## Arrhythmias

As more attention is paid to cardiotoxic side effects, more and more oncologic treatment regimens are being linked to a wide variety of electrophysiological changes and arrhythmias [78]. Although associated with an increased overall long-term mortality, arrhythmias related to chemotherapy are mostly transient and not life-threatening [79]. The diagnostic workup should include a 12 lead ECG, Holter monitoring, echocardiography and a laboratory workup with serum electrolytes and renal function tests.

Chemotherapy-induced arrhythmia has been documented for anthracyclines, taxanes, and cyclophosphamide. A short infusion duration of anthracyclines increases the likelihood for transient supraventricular arrhythmia and ventricular premature beats (VPB) following administration [80]. Taxane therapy might lead to sinus bradycardia and first-degree atrioventricular block in some patients, which usually do not require any further therapy [81]. Up to 10% of all patients receiving high-dose cyclophosphamide develop supraventricular or ventricular arrhythmia within the first 3 days after administration [78, 82]. Patients with perimyocarditis or other chemotoxic side effects are at increased risk [78, 82].

In addition, many chemotherapeutic drugs can lead to QT-prolongation. Chemotoxic side effects like diarrhea or vomiting as well as antiemetic drugs and psychotropic medication can enhance QT-prolongation [82]. Susceptibility to torsades de pointes tachycardia with potential conversion to ventricular fibrillation depends on the chemotherapeutic drug used [83–85]. Therefore, there is no general recommendation concerning a QTc-interval, at which chemotherapy should be suspended [85].

While the above-mentioned transient arrhythmias are mostly direct side effects and resolve when chemotherapy is suspended, chronic or recurrent arrhythmias are often due to structural myocardial damage caused by chemotherapeutic agents (e.g., anthracyclines) [80]. Myocardial scarring of the cardiac conduction system caused by chest radiotherapy leads to atrioventricular blocks [86, 87]. Rarely, myocardial tumor infiltration or pericardial metastasis might be the cause of arrhythmia, which are usually associated with a poor prognosis and should be treated symptomatically.

Beside arrhythmias, recent data indicate that resting heart rate alone could serve as a predictive marker for patients with colorectal, pancreatic, and non-small cell lung cancer [88].

## Conclusion

The challenge of cardio-oncology is getting the right diagnosis from merely unspecific symptoms in order to balance the urgent and often life-saving oncologic therapy against cardiotoxic side effects limiting quality of life and long-term survival. Thus, a close collaboration between oncologists/hematologists and cardiologists is crucial. Cancer patients should, therefore, be seen by a cardiologist before, during and after a potentially cardiotoxic chemotherapy. Further research is needed to improve our understanding of cancer-related cardiovascular risks and to develop parameters for a better cardiovascular risk stratification of cancer patients.
